# NNK-Induced Lung Tumors: A Review of Animal Model

**DOI:** 10.1155/2011/635379

**Published:** 2011-04-27

**Authors:** Hua-Chuan Zheng, Yasuo Takano

**Affiliations:** Kanagawa Cancer Center, Clinical Research Institute, Yokohama 241-0815, Japan

## Abstract

The incidence of lung adenocarcinoma has been remarkably increasing in recent years due to the introduction of filter cigarettes and secondary-hand smoking because the people are more exposed to higher amounts of nitrogen oxides, especially 4-(methylnitrosamino)-1-(3-pyridyl)-1-butanone(NNK), which is widely applied in animal model of lung tumors. In NNK-induced lung tumors, genetic mutation, chromosome instability, gene methylation, and activation of oncogenes have been found so as to disrupt the expression profiles of some proteins or enzymes in various cellular signal pathways. Transgenic animal with specific alteration of lung cancer-related molecules have also been introduced to clarify the molecular mechanisms of NNK in the pathogenesis and development of lung tumors. Based on these animal models, many antioxidant ingredients and antitumor chemotherapeutic agents have been proved to suppress the NNK-induced lung carcinogenesis. In the future, it is necessary to delineate the most potent biomarkers of NNK-induced lung tumorigenesis, and to develop efficient methods to fight against NNK-associated lung cancer using animal models.

## 1. Introduction

Throughout the spectrum of cancers worldwide, lung cancer claims the lives of over one million people worldwide each year and is one of the most common and lethal cancers of men and women in North America, Europe, and East Asia although current strategies in the treatment of lung cancer including surgery, radiation therapy, chemotherapy, and targeted biological therapies have slightly generated an increase in the 5-year survival rate for all stages combined [1]. The secular trend in lung cancer histology indicates that the proportion or incidence of lung adenocarcinoma has been increasing markedly over the past two decades, surpassing the squamous cell carcinoma as the most common histological subtype of lung cancer in many countries, which is partly due to the introduction of filter cigarettes and secondary-smoking because the people are more exposed to higher amounts of nitrogen oxides, nitrosated compounds, and lung-specific smoke carcinogens [1–3]. 

Epidemiological and laboratory evidences demonstrate a strong etiological association with smoking, which contains volatile N-nitrosamines such as N-nitrosodimethylamine, N-nitrosopyrrolidine as well as tobacco-specific N-nitrosamines such as N′-nitrosonornicotine and 4-(methylnitrosamino)-1-(3-pyridyl)-1-butanone (NNK). Although N′-nitrosonornicotine causes tumors of the oesophagus and nasal cavity in rats, NNK reproducibly induces pulmonary adenocarcinomas (PAC) in laboratory rodents, including rats, mice, hamsters, and ferrets, which therefore has been classified as a human lung carcinogen by the International Agency for Research on Cancer working group. Significant incidences of tumors occurred in the lungs of strain A/J progeny (24 wk) and in the livers of male C3B6F1 and Swiss progeny (72 wk) after NNK treatment [4]. NNK is known to be activated in the lung via *α*-carbon hydroxylation by cytochrome P450 (CYP), hemoglobin, and lipoxygenases (LOX) [5]. The production of methylating and pyridyloxobutylating agents that attack DNA and cause the genetic changes is known to be associated with self sufficiency in growth signals, evasion of apoptosis, insensitivity to antigrowth signals, sustained angiogenesis, tissue invasion and metastasis, and limitless replicative potential.

With lung carcinogenesis models, it may be helpful to gain insights into basic biology of lung tumors, find out markers for early diagnosis, and validate antilung cancer prevention and therapies. Ferrets exposed to both NNK and smoke developed preneoplastic lesions (squamous metaplasia, dysplasia, and atypical adenomatous hyperplasia) with complex growth patterns and further exposure will cause squamous cell carcinoma, adenosquamous carcinoma, and adenocarcinoma [6, 7]. In A/J mice, 14-week NNK treatment can cause pulmonary hyperplasias along the alveolar septa, in which the proliferating cells showed cuboidal shape, lamellar bodies, and centrally localized ovoid nuclei as type II pneumocytes. From 34 to 42 wks after treatment, progression to neoplasia was characterized by a declined hyperplasias and an increased adenoma. Carcinomas appeared to increase in frequency 34 wks after NNK treatment and comprised more than 50% of the pulmonary lesions by 54 wks. The growth pattern of carcinomas began to change from solid to mixed (solid and papillary) 42 wks after NNK treatment [8].

## 2. Mechanisms of NNK-Induced Lung Carcinogenesis

The establishment of genetic and epigenetic alteration followed by gene expression profiling is of great use and help to clarify molecular mechanisms of NNK-induced lung tumorigenesis. In experimental model, NNK could cause Adrb2 SNP mutation of Syrian golden hamsters, and K-ras mutation in codon 12 of the A/J mice [9–11]. Bacterial artificial chromosome array-based comparative genomic hybridization indicated that the gains on chromosomes 6 and 8, and losses on chromosomes 11 and 14, were more common in NNK-induced tumors and the changes on chromosomes 8, 11, 12, and 14 were positively associated with the degree of chromosome instability [12, 13]. The methylation of the retinoic acid receptor (RAR)-*β* and death-associated protein kinase gene was also detected in preneoplastic hyperplasias or adenocarcinoma induced by NNK treatment [14–16].

NNK administration reduced the miR-126* expression targeting CYP2A3 in rat lungs, but induced CYP2A3 expression [17]. The 14-3-3 isoforms (*θ*, *ξ*, and *σ*) and annexin A5 were significantly downregulated in NNK-induced pulmonary adenocarcinogenesis of A/J mice according to 2D-electrophoresis [18]. Immunohistochemically, NNK induced preneoplastic lesions in lungs, including alveolar hyperplasia and atypical dysplasia with COX-2 and PCNA overexpression [19]. In lung adenocarcinoma of hamsters, the overexpression of PKA, cAMP, CREB and phosphorylated CREB in *β*2-adrenergic receptor pathway and EGFR-specific phosphorylated tyrosine kinase, Raf-1 and ERK1/2 and their phosphorylated forms in EGFR pathway were observed [20]. Regarding the control of cell cycle, there was an upregulated expression of cyclin D1 and cdk4, but downregulated Rb expression in NNK-induced lung adenomas and adenocarcinomas [21]. Exposure of NNK to hamsters and mice led to the decreased expression of Clara cell 10-kDa protein (CC10) which subsequently enhances the induction of anchorage-independent growth in response to NNK [22]. The NNK treatment enhanced the expression of fatty acid synthase, transketolase, pulmonary surfactant-associated protein C, L-plastin, annexin A1, and haptoglobin, but the expression of transferrin, *α*-1-antitrypsin, and apolipoprotein A-1 decreased [23]. The NNK-mediated expression of protein and RNA in mouse lung tumors will provide more information and clues about markers of NNK-induced tumroigenesis and targets for antipreventive agents in NNK-related lung cancer.

## 3. Transgenic Animal Model of NNK-Mediated Lung Carcinoma

Transgenic models have the potential to play an important role in identification of potential human carcinogens and clarify the molecular mechanisms of carcinogens in the pathogenesis and development of malignancies. Actually, many transgenic and knockout mice have been applied to investigate the NNK-induced lung carcinogenesis. 

When transgenic mice that overexpress HGF in the airway epithelium were exposed to NNK, they exhibited congestion in the alveolar spaces, excessive production of blood vessels, a convoluted pattern of airways with more number of lung tumors, and high tumor incidence, compared with control [24]. All NNK-treated SPC/myc transgenic mice showed bronchioloalveolar hyperplasia and adenocarcinoma formation [25]. The p53 mutation, on an A/J F1 background, were more susceptible to NNK and mice with a mutant p53 developed larger lung tumors, emphasizing the potential effects of a p53 mutation both on tumor initiation and progression [26]. NNK-treated mice expressing high levels of IGF-IR transgene developed larger tumors than the control mice [27]. 

 Galectin-3, a *β*-galactoside-binding lectin is a multifunctional protein, which regulates cellular adhesion, proliferation, and apoptosis, and in turn contributes to tumorigenesis. We intraperitoneally administrated NNK into galectin-3 wild-type (gal3+/+) and knockout (gal3−/−) mice and found that the incidence of lung tumors was significantly lower in gal3−/− than gal3+/+ mice after 32 wks. Compared with gal3+/+ mice, pathway analysis of gene microarray indicated that galectin-3 upregulated carcinogenesis-related genes (e.g., B-cell receptor, ERK/MAPK, and PPAR signalings) in normal condition, and NNK-induced gene expression were associated with cellular growth (e.g., Wnt/*β*-catenin signaling) or immunological disease (e.g., EGF and PDGF signalings) in lung carcinogenesis regardless of galectin-3 status. The functions involved in NNK-induced PAC include cellular growth and proliferation and canonical pathways for Wnt/*β*-catenin signaling [28, 29].

Human methylguanine-DNA methyltransferase (MGMT) transgenic mice overexpressing O^6^-alkylguanine-DNA alkyltransferase (AGT) in lung were crossbred with A/J mice for animal model of lung tumors. The MGMT transgenic mice had higher AGT activity, lower multiplicity, and smaller-sized lung tumors than the control mice after NNK treatment. Moreover, a reduction in K-ras mutations in lung tumors was found in the MGMT transgenic mice [30]. When MGMT−/− mice were crossed with a lacI-based transgenic reporter line, NNK-dependent lacI mutations was more frequently observed in MGMT−/− tissues. The mutational spectra of NNK-treated MGMT−/− lungs revealed an increase in G:C to A:T changes accompanied by a shift from CpG to GpG sites [31]. 8-hydroxyguanine DNA glycosylase 1 (Ogg1) gene encodes an enzyme that repairs an oxidative DNA injury 8-oxoguanine (8-oxoG), whose deficiency results in the development of lung adenomas and preneoplastic atypical hypreplasias in knockout mice treated with NNK [32].

There was a NNK-dose-dependent increase in lung tumor size in PTEN+/−, compared with +/+ mice. Lung tumors from PTEN+/− mice had K-ras mutations, low PTEN expression, and Akt pathway activation [33]. Although mice with a knockout of G-protein coupled receptor 5A develop lung tumors after a long latent period, NNK treatment could hurry the development of lung tumors, exhibiting increased tumor incidence and multiplicity and a dramatic increase in lesion size [34]. The 4 eukaryotic initiation factor (ebp)1−/−/4ebp2−/− mice showed increased sensitivity to NNK-induced tumorigenesis, compared with the wild-type counterparts due to translational activation of genes governing angiogenesis, growth and proliferation and translational activation of CYP2A5 [35]. NNK exposure of CC1-knockout mice causes a significantly higher incidence of airway epithelial hyperplasia and lung adenomas with K-ras mutation, Fas ligand expression and MAPK/ERK phosphorylation increased than wild-type littermates [36].

The numbers of NNK-induced lung tumors and tumor multiplicity were reduced in the lung-NADPH-P450-reductase (Cpr)-null mice, relative to wild-type mice, which was correlated with lower lung O6-methylguanine adduct levels. Lung tumors in lung-Cpr-null mice were positive for CPR expression, indicating that the tumors did not originate from Cpr-null cells [37]. With the NNK treatment, the tumor multiplicity in angiotensin II type 2 receptor (AT2)-null mice was significantly smaller than that in wild-type mice [38].

## 4. Repressing of NNK-Induced Lung Carcinogenesis

The antioxidants (Selenium, *β*-carotene, N-acetylcysteine, and *α*-tocopherol) from tea, plant, or vitamin can prevent lung carcinogenesis in an NNK-treated ferret model by preventing oxidative DNA damage, and increasing the levels of lung retinoic acid in the lung cancer induced by NNK. Chemotherapeutic agents also play a preventive role in the NNK-induced lung tumorigenesis due to the modification of disrupted signal pathways. 

### 4.1. Selenium

1,4-phenylenebis(methylene)selenocyanate (p-XSC) was highly effective to inhibit the initiation and postinitiation phase of lung tumorigenesis induced by NNK in A/J mice and reduce NNK-induced DNA methylation and 8-hydroxy-2′-deoxyguanosine (8-OHdG) levels in the lung [39]. The levels of protein-bound:free GSH ratios and Cys ratios were significantly decreased, and total glutathione S-transferase (GST) enzyme activity, as well as GST-pi and GST-mu enzyme activities, glutathione peroxidase (GPX) activity were significantly induced in p-XSC-treated mice after NNK treatment. These results suggest that p-XSC inhibits tumor formation partially by protecting against oxidative damage [40]. Additionally, p-XSC was shown to significantly inhibit formation of O6-methylguanine and 7-methylguanine in the mouse lungs treated with NNK, indicating its inhibitory role in DNA methylation [41]. Additionally, 2-oxo-selenazolidine-4(R)-carboxylic acids and selenocystine significantly reduced lung adenoma multiplicity in NNK-treated mice with hepatic selenium levels elevated [42].

### 4.2. Tea

The inhibitory effect of tea on lung carcinogenesis has been attributed to its major ingredients, such as polyphenolic compound, epigallocatechin gallate (EGCG), caffeine, thearubigins, and theaflavins because tea inhibited the formation of reactive oxygen species and radicals, and induced CYP1A1, 1A2 and 2B1, and glucuronosyl transferase [43]. In NNK-induced lung tumors, tea treatment inhibited angiogenesis, as indicated by the lower microvessel density and enhanced the apoptosis index labeled by TUNEL [44]. The levels of 8-hydroxydeoxyguanosine, a marker of oxidative DNA damage, were significantly suppressed in NNK-induced mice treated with green tea or EGCG. The oxidation products found in black tea, thearubigins, and theaflavins, also possessed antioxidant activity and retarded the development of lung cancer caused by NNK [45]. The administration of Polyphenon E and Caffeine not only reduced the incidence and multiplicity of lung adenocarcinoma in female A/J mice induced by NNK, but also inhibited cell proliferation, enhanced apoptosis, and lowered levels of c-Jun and ERK1/2 phosphorylation in adenocarcinomas and adenomas, suggesting that tea polyphenols (and perhaps caffeine) inhibit NNK-induced lung tumorigenesis [46].

### 4.3. Vitamin

Vitamin E inhibits tumor cell growth in vitro irrespective of its antioxidative effect. In NNK-induced lung tumors of mice, Vitamin E supplement reduced the mutation frequency of K-ras at codon 12, suggesting that it suppresses NNK-induced DNA injury [47]. *α*-tocopheryloxybutyric acid (TSE), a nonantioxidative vitamin E derivative, could inhibit cell proliferation during the mouse lung tumorigenic process treated with NNK. The administration of Vitamin E or TSE suppressed the labeling index of the PCNA, the elevation of ornithine decarboxylase activity at a promotion phase of NNK-induced lung tumorigenesis [48, 49]. *γ*-tocopherol-rich mixture of tocopherols (*γ*-TmT, considered as vitamin E) significantly lowered tumor multiplicity, tumor volume, and tumor burden, which was associated with high apoptosis and low levels of 8-hydroxydeoxyguanine, *γ*-H2AX and nitrotyrosine in the NNK-induced lung [50].

 Mice receiving the supplementation of 1*α*,25-dihydroxyvitamin D3 (1,25D) had significantly lower tumor incidence and tumor multiplicity, but experienced body weight loss, kidney calcium deposition, elevated kidney CYP24 expression, and decreased fasting plasma 1,25D levels [51]. Inhaled mid-dose isotretinoin (13-cis-retinoic acid) caused up-regulation of lung tissue nuclear RARs relative to vehicle-exposed mice [52]. 9-cis-Retinoic acid (9cRA) binds both RARs and retinoid X receptors and has been shown to be a potential chemopreventive agent. The mice receiving 9cRA supplementation had significantly lower tumor multiplicity and showed a trend toward lower tumor incidence, as compared with the mice given NNK alone [53]. The mice exposed to the high isotretinoin (13-cis retinoic acid) dose or 5-hydroxy-4-(2-phenyl-(E) ethenyl)-2(5H)-furanone (KYN-54, a novel retinoidal butenolide compound) showed reductions of tumor multiplicity after NNK treatment [54].

### 4.4. Plant and Vegetable

The discovery of dietary-related compounds with potential to inhibit lung cancer may present promising and practical approaches to reduce the risk of lung cancer caused by smoking. The exposure to fermented brown rice and rice bran significantly reduced the multiplicity, and tumor size of NNK-induced lung tumor with the expression of CYP 2A5 mRNA and Ki-67 protein decreased [55]. Deguelin, a natural plant product, specifically inhibits the proliferation of premalignant and malignant bronchial epithelial cells by blocking Akt activation [56]. Feeding with powdered adlay seed exerts an anticancer effect, evidenced by the reduced number of surface lung tumors [57]. The administration of Changkil saponins suppressed the NNK-induced increase in the level of PCNA and the number of lung tumors [58]. The treatment of 7-hydroxy-3-methoxycadalene from Zelkova serrata significantly reduced the incidence of adenomas and adenocarcinoma in a concentration-dependent manner [59]. Cinnamaldehyde (CNMA) treatment significantly reduced the combined incidence of adenomas and carcinomas, tumor multiplicity in transgenic rasH2 male mice [10]. Kava is a traditional beverage in the South Pacific islands and could prevent NNK plus BaP-induced lung tumorigenesis in A/J mice by enhancing apoptosis, inhibiting proliferation and the activation of NF-kappaB in lung tumors [60]. Isothiocyanates are derived from cruciferous vegetables and their N-acetylcysteine and phenethyl conjugates inhibit the formation of lung adenoma and adenocarcinoma with a significant reduction in PCNA and an induction of apoptosis in A/J mice induced by NNK [61, 62]. In (A/J × TSG-p53 “knockout”) F1 mice with either the p53+/− or p53+/+ genotype, phenethyl isothiocyanate (PEITC) pretreatment significantly decreased tumor incidence and multiplicity [63]. *β*-carotene increased lung tumor multiplicity, lung tumor size, blood cell cAMP, serum, and lung levels of retinoids and induced p-CREB and p-ERK1/2 in PAC induced by NNK [64]. Treatments with Satsuma mandarin juice (MJ), MJ2, and MJ5 reduced the incidence and multiplicity of NNK-induced lung tumors by decreasing PCNA-positive index in lung tumors [65].

### 4.5. Enzyme Inhibitor

CYP enzymes can catalyze the *α*-hydroxylation of NNK for its activation in the oxidative metabolism pathway, such as CYP2A6. A trend was noted for 8-Methoxypsoralen (8-MOP), an inhibitor of CYP2A6, to reduce adenomas and adenocarcioma to a greater extent than hyperplasia in mouse lung treated by NNK [66–69]. NNK is reported to promote COX-2 activity in colon and gastric cancer cells and the development of NNK-induced adenocarcinomas in mice is reduced by inhibitors of cyclooxygenase [70]. Another report showed that such specific COX-2 inhibitors as acetylsalicylic acid or N-[2-(cyclohexyloxy)-4-nitrophenyl]-methanesulfona-mide significantly increased the apoptotic index and inhibited the expression of COX-2 in NNK-treated mice [71]. Farnesyltransferase inhibitors (FTIs) included manumycin, gliotoxin, dihydroepiandrosterone, perillyl alcohol, and FTI-276. FTI-276 reduced both the tumor multiplicity and the total tumor volume/burden per mouse. The apoptotic index in FTI-276-treated tumors showed an increase of 77% over control tumors [72].

### 4.6. Fatty Acid

The supplementation of fish oil with a low *ω*-6 (n-6)/*ω*-3 (n-3) polyunsaturated fatty acid ratio was able to significantly decrease lung tumor prevalence compared to groups receiving soybean oil and corn oil, which was associated with increased expression of cell cycle inhibitor p21Cip1 and lipoxygenase isoform 15-LOX in the lungs [73]. The treatment of NNK increased the level of prostaglandin E2 as well as PCNA and induced the activation of an ERK cascade (ERK, MEK, and Raf-1) in high linoleic acid oil- (LA-) fed mice. On the other hand, oleic acid oil (OA) feeding abolished the NNK-induced activation of the ERK cascade. In conjugation with these events, OA feeding reduced lung tumor incidence and tumor multiplicity in mice compared with LA feeding. These results suggest that OA suppresses lung tumorigenesis and that this suppression is correlated with the inhibition of PGE2 production and inactivation of the ERK cascade [74]. Myoinositol in AIN-93 diet also proved to reduce the development of lung tumors induced by NNK [75].

### 4.7. Anticancer Reagents

The anticancer chemicals targeting the cell signals and metabolism can be employed to prevent carcinogenesis. Gefitinib is an EGFR tyrosine kinase inhibitor (EGFR-TKI) and could significantly suppress the multiplicities of the NNK-induced tumors in a dose-dependent manner [76]. The administration of Rapamycin, an inhibitor of mTOR, decreased tumor size, proliferative rate, tumor multiplicity, and mTOR activity in NNK-treated mice [77]. Targretin is a retinoid specifically selective for retinoid X receptors and widely used as an anticancer reagent. In mice, it could decrease the multiplicity and size of NNK-induced tumors, demonstrating its preventive and therapeutic activity [78]. Histone deacetylase inhibitors, such as suberoylanilide hydroxamic acid (SAHA), showed a significant inhibition of lung tumor multiplicity in mice treated with NNK. However, a significant inhibition of the alpha-hydroxylation pathway of NNK was observed in lung microsomes, suggesting that SAHA may act to inhibit the activation pathways of NNK metabolism [79].

## 5. Future Perspectives

The contribution of NNK to the imbalance between cellular proliferation and apoptosis, and subsequent lung tumorigenesis has been consistently described and confirmed in numerous animal models. According to animal experiments, several mechanisms of NNK-induced lung carcinoma have been proposed, including (i) the activation of oncogenes via mutation, (ii) interruption and/or silencing of genes encoding enzymes coupled with NNK, (iii) direct manipulation of enzymes (specifically from the CYP protein family) responsible for activation and initiation of NNK-mediated processes, and (iv) the disruption of the signal pathways. Some primary preventive approaches have not yet been established, including (i) rendering NNK inactivation by antioxidants (tea, vegetable, vitamin and metal compunds) and (ii) obstructing the function of NNK (anticancer chemotherapeutic agents). In the recent years, too many transgenic mice has been bred and applied in the establishment of lung cancer model, which can provide an efficient tool for the investigation of lung cancer without the influence of chemical carcinogen. If treated with NNK, it is of great help and use to clarify the molecular mechanism of NNK-induced lung carcinogenesis and find out the novel target to prevent NNK-associated lung cancer. Therefore, it is necessary to delineate the most potent biomarkers of NNK-induced lung tumorigenesis, and to develop efficient methods to fight against this kind of lung cancer using animal model in the future.
